# Lysine and homoarginine are closely interrelated metabolites in the rat

**DOI:** 10.1007/s00726-022-03158-0

**Published:** 2022-03-30

**Authors:** Svetlana Baskal, Laurianne Dimina, Stefanos A. Tsikas, Laurent Mosoni, Didier Remond, François Mariotti, Dimitrios Tsikas

**Affiliations:** 1 Institute of Toxicology, Core Unit Proteomics, Hannover Medical School, Carl-Neuberg-Str. 1, 30623 Hannover, Germany; 2 Université Paris-Saclay, AgroParisTech, INRAE, UMR PNCA, 75005 Paris, France; 3 Academic Controlling, Hannover Medical School, 30623 Hannover, Germany; 4 Université Clermont Auvergne, INRAE, UNH, Clermont-Ferrand, France

**Keywords:** AGAT, L‐Arginine, Arginase, Guanidinoacetate, GC–MS, L‐Homoarginine, L-Lysine, Metabolism, Plasma

## Abstract

L-Lysine (Lys) and L-arginine (Arg), but not L-homoarginine (hArg), are proteinogenic amino acids. In healthy humans, oral administration of hArg increased the plasma concentration of Lys, suggesting Lys as a metabolite of hArg. In humans and animals, hArg is biosynthesized from Arg and Lys by arginine:glycine amidinotransferase (AGAT). In vitro, recombinant human arginase and bovine liver arginase I hydrolyzed hArg to Lys, suggesting Lys as a metabolite of hArg. The aim of the present study was to investigate whether changes in blood concentrations of hArg and Lys in old rats fed for 4 months with varied controlled experimental diets could suggest interconversion of these amino acids. Blood samples (*n* = 253) were taken before (T0) and after 2 months (T2) and 4 months (T4) of the experiment. Plasma concentrations of Lys and hArg were determined by gas chromatography–mass spectrometry. The plasma hArg concentration markedly correlated with the plasma Lys concentration at all timepoints (*r* ≥ 0.7, *P* < 0.0001). Further analysis demonstrated that hArg and Lys are closely and specifically associated independently of experimental time/rat age and diet, suggesting that hArg and Lys are mutual metabolites in old rats. Based on the plasma concentration changes, the median yield of hArg from Lys was determined to be 0.17% at T0 and each 0.27% at T2 and T4. With a circulating concentration of about 3 µM, hArg a major metabolite of Lys in healthy humans. hArg supplementation is currently investigated as a cardioprotective means to improve impaired hArg synthesis. Present knowledge suggests that Lys rather than hArg supplementation may be even more favorable.

**Supplementary Information:**

The online version contains supplementary material available at 10.1007/s00726-022-03158-0.

## Introduction

**Fig. 1:**
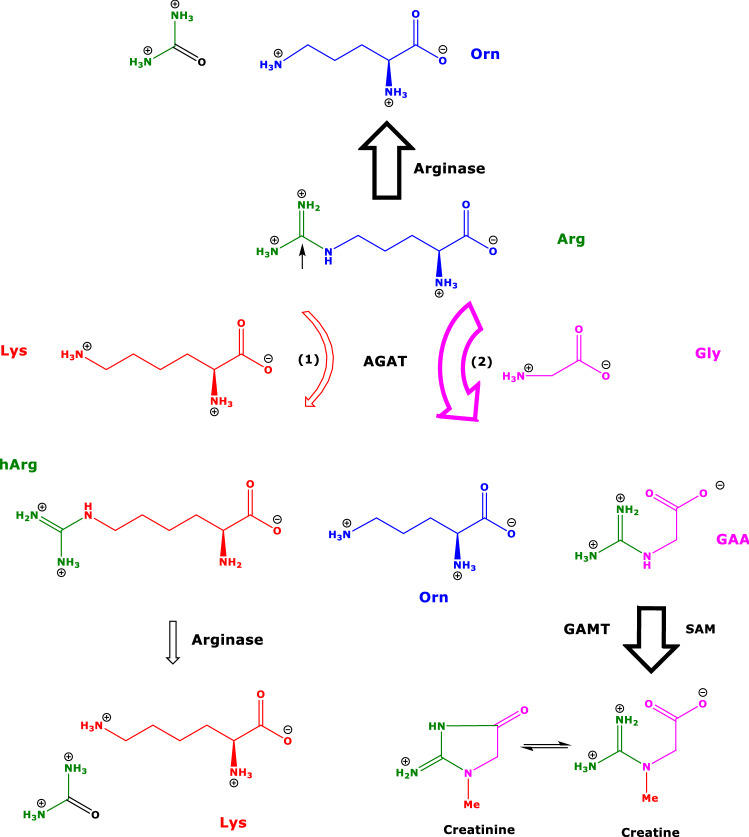
Simplified schematic of the arginine:glycine amidinotransferase (AGAT)-catalyzed biosynthesis of (1) L-homoarginine (hArg) from L-arginine (Arg) and L-lysine (Lys), and (2) of guanidinoacetate (GAA) from Arg and glycine (Gly), with L-ornithine (Orn) being the second common reaction product. Arginase hydrolyzes hArg to Lys and urea. GAA is methylated by guanidinoacetate *N*-methyltransferase (GAMT) to creatine using *S*-adenosylmethionine (SAM) as the methyl (Me) group donor. Creatine cyclizes to creatinine. The arrow below the structure of Arg indicates the C atom that is involved in the catalytic process. The width and thickness of the arrows indicates roughly the extent of the reactions

In a pioneering work, injection of ^14^C-labelled L-lysine together with unlabeled L-lysine (15 mmol/kg bodyweight) in rats resulted into formation of ^14^C-labelled homoarginine (^14^C-hArg); ^14^C-hArg was found in the liver and in the kidney of the rats (Ryan and Wells 1964). Ingestion of 4 g of Lys·HCl by healthy humans resulted in the excretion in the urine of hArg, which was not detectable before Lys·HCl ingestion (Ryan and Wells 1964). A few years later, [^14^C-*guanidino*]hArg was found to be metabolized to ^14^C-urea by the isolated perfused rat liver, presumably by the action of liver arginase (Ryan et al. 1968). The enzyme arginine:glycine amidinotransferase (AGAT; EC 2.1.4.1) catalyzes at least two L-arginine-involving reactions: (1) the formation of L-homoarginine (hArg) from L-arginine (Arg) and L-lysine (Lys); and (2) the formation of guanidinoacetate (GAA) from Arg and glycine (Gly) (Tsikas and Wu 2015) (Fig. 1). It is worth mentioning that AGAT catalyzes the transfer of guanidine groups from several donors to different acceptors in various organs including the kidney and the pancreas (Srivenugopal and Adiga 1980; Watanabe et al. 1988, 1994). GAA is methylated to creatine by guanidinoacetate *N*-methyltransferase (GAMT; EC 2.1.1.2) using *S*-adenosyl-L-methionine (SAM) as the methyl group donor. Creatine is further converted to creatinine.

hArg is not a proteinogenic amino acid. hArg and GAA are not present in proteins including human serum proteins (Bollenbach et al. 2018). In humans, hArg and GAA circulate in blood and are excreted in the urine. The concentrations of GAA and hArg are comparable in human blood (molar ratio about 3:1). In human urine, however, GAA and hArg differ greatly (molar ratio about 100:1). Considering that creatinine is a final metabolite of GAA, the whole body synthesis of hArg in humans appears very low compared to the biosynthesis of GAA. This suggests that the hArg-producing AGAT activity (reaction 1; Fig. 1) is much lower than the GAA-producing activity of AGAT (reaction 2; Fig. 1) (Tsikas and Wu 2015).

Low circulating and low excretory concentrations of hArg are associated with worse cardiovascular outcome and mortality (Pilz et al. 2015; Atzler et al. 2015; Frenay et al. 2015; Kayacelebi et al. 2017; Zinellu et al. 2018). Impaired hArg homeostasis might contribute to renal, cardiovascular and cerebrovascular pathophysiology. Even though hArg can exert nitric oxide (NO)-dependent physiological functions (Tsikas et al. 2018), the underlying mechanisms are largely elusive. Nevertheless, supplementation of hArg is considered to be of pharmacological importance in conditions associated with impaired hArg synthesis (Atzler et al. 2016; Schonhoff et al. 2018; Zinellu et al. 2018).

The metabolic fate of hArg in humans is insufficiently investigated although pioneering studies have been reported several decades ago (Ryan and Wells 1964; Ryan et al. 1968). Due to the emerging importance of hArg, several studies investigated more recently the metabolism of hArg in humans. hArg was demonstrated to be oxidized to 6-guanidino-2-oxocaproic acid by alanine:glyoxylate aminotransferase 2 (AGXT2; EC 2.6.1.44) (Rodionov et al. 2016). In human plasma, the concentration of 6-guanidino-2-oxocaproic acid is about 1000 times lower than that of hArg (Martens-Lobenhoffer et al. 2018), suggesting 6-guanidino-2-oxocaproic acid as a minor metabolite of hArg. Arginases hydrolyze very rapidly Arg to L-ornithine (Orn) and urea. By contrast, recombinant human arginase and isolated bovine liver arginase I hydrolyze hArg to Lys very slowly (Bollenbach et al. 2019), suggesting that Lys is a minor arginase–metabolite of hArg (Fig. 1). Ornithine decarboxylase (ODC) converts hArg to homoagmatine (Tsikas et al. 2020). As homoagmatine has not been found in humans thus far, its concentration is considered much lower than that of agmatine, which is produced by arginine decarboxylation of the remarkably more abundant Arg. In healthy humans, Lys was found to be a metabolite of supplemented hArg (Bollenbach et al. 2019).

Previous studies have investigated the metabolism of supplemented Lys to hArg, or of supplemented hArg to Lys. Demonstration of the conversion of non-radiolabeled hArg to Lys in humans is very challenging, because the concentration of hArg in blood is about 100 times lower than that of Lys (Yamamoto et al. 2016; Bollenbach et al. 2019). Nevertheless, ingestion of relatively large amounts of Lys, hArg or biochemically closely related amino acids such as Arg may cause metabolic imbalances (Tews and Harper 1986; Yang et al. 2015; Hou et al. 2016; Günes et al. 2017). Low protein diet (2.5% protein) supplemented with Lys (2.5% Lys·HCl) to young (3.25 months) male Sprague–Dawley rats for 1.25 months resulted in increases of plasma concentrations of Ala, Pro, Arg and hArg (Shimomura et al. 2014).

The aim of the present study was to investigate whether endogenous Lys and hArg are mutual substrates in the rat and whether their homeostasis vary according to experimental time/rat age and diet in a medium/long term experimental setting. For this purpose, we measured free Lys, hArg and many other amino acids in plasma samples of rats repeatedly in experimental conditions over 4 months and studied the associations between the amino acids. Free metabolites of Lys and Arg from enzymatic and non-enzymatic post-translational modifications (PTM) of these amino acids were also measured.

## Materials and methods

### Rats study

Male Wistar rats (15 months, 96 rats at the beginning, 77 rats at the end of the study) were habituated to housing for 3 months in our animal facility (two animals per cage, enrichment in the cage, temperature 22 °C, inverted light cycle 12 h/12 h, ad libitum feeding). They were given free access to water and commercial laboratory chow (SAFE® A04, site de fabrication des aliments, 89290 Augy, France) containing 16% protein, 3% fat, 60% carbohydrates, water, fibers, vitamins and minerals according to AIN93 recommendations for maintenance (Reeves et al. 1993). During the habituation period, animals were fasted overnight and basal measurements such as blood withdraw, body weight and composition were conducted. Blood was collected in the fasted state from a lateral tail vein. The baseline point is referred to as T0 and corresponds to the start of the experiment. At the end of the habituation period, the rats were randomly divided into four groups of identical average weight and average lean mass, and each group was fed for 4 months either standard or high-saturated/fat-high sucrose kibbles differing according to the protein source (milk- or plant-based). Measurements were repeated 2 months (T2, rats aged 20 months) and 4 months (T4, rats aged 22 months) after the beginning of the long-term controlled conditions including experimental diets. Animals were handled according to the recommendations of the Regional Review Committee (approval of application number: APAFIS# 23389-2019122010536779 v2). All procedures were in accordance with the guidelines formulated by the European Community for the use of experimental animals (L358–86/609/EEC, Council Directive, 1986).

### GC–MS quantification of amino acids in rat plasma samples

We measured amino acids in plasma samples of all animals at baseline (T0), after 2 months (T2) and after 4 months (T4) supplementation with four different diets. The concentration of free amino acids and some of their metabolites in plasma samples (10 µL) was determined by previously reported stable-isotope dilution gas chromatography–mass spectrometry (GC–MS) methods (Hanff et al. 2019; Baskal et al. 2021). The methods involve use of de novo prepared trideuteromethyl esters of the amino acids as internal standards, esterification and *N*-pentafluoropropionylation. Aliquots (1 µL of toluene extracts) were injected splitless in the GC–MS apparatus model ISQ from ThermoFisher (Dreieich, Germany). Amino acids were analyzed in the negative-ion chemical ionization mode by monitoring selected ions for endogenous amino acids and the respective internal standards as described previously (Hanff et al. 2019; Baskal et al. 2021). The dwell time was 100 ms for each ion. The peak areas of the amino acids and their respective internal standards were used for quantification. For methodological reasons (Hanff et al. 2019) the following amino acids were measured and reported as their sum: Leu and Ile, Asp and Asn, Glu and Gln, Orn and Cit. We observed two 5-hydroxy-lysine metabolites (Baskal et al. 2021) and considered them separately in all analyses. The percentage molar ratio of plasma hArg to plasma Lys was used as a measure of the yield of hArg from Lys.

### Statistical analyses

Statistical analyses were performed on the full data set, which consisted of 32 targeted metabolites (Supplement, Table S1) measured in 253 samples and obtained at 3 timepoints.

Correlation analysis was used to estimate the all-pairwise correlations (Pearson’s *r* and *P* value for independence) between the metabolites and to identify which metabolites were correlated with hArg. Correlation analysis was performed considering all timepoints together and then each timepoint separately. Correlations were considered high for |r|> 0.7.

After time effect correction (Karpievitch et al. 2015), partial correlation networks were built to represent the correlations between the metabolites across all timepoints. A data-driven network approach was used implementing the de-biased sparse partial correlation (DSPC) algorithm (Basu et al. 2017).

Univariate analyses were conducted using one-way ANOVA, post-hoc Fisher’s least significant differences (LSD) test and regression analysis between time groups. Data are reported as means ± standard deviation for normally distributed data and as median with interquartile range (IQR) otherwise.

Partial least squares (PLS) regression was used with hArg as Y-response factor and all metabolites as X-predictors using NIPALS algorithm. It was used as a tool for finding a few underlying predictive factors that account for most of the variation in the response. Regression coefficient profile and plot showing variables of importance were used as a direct indication of which variables are most useful for predicting hArg. Metabolites with a value of VIP > 0.8 and a relatively high score were considered as explanatory.

A selection algorithm using the stepwise selection method (based on the SBC criterion) was used to identify a parsimonious ANOVA model that explain hArg with selected metabolites and time as a class variable. The relative importance of the selected metabolites in the model was evaluated with the standardized coefficients. Cross validation was used to assess the predictive performance of the model (with the "leave-one-out" cross validation procedure).

We also ran a higher statistical model to test whether the association between Lys and hArg could have been affected by the characteristic of the diets, especially including the proteinic Lys of the diets. The Lys content (in mg/kg) was 7200 for habituation diets, 12,570 for high fat milk-based diet, 7020 for high fat plant-based diet, 10,470 for standard milk-based diet, and 5860 for standard plant-based diet. Lys intake, taken as a cofactor in the model, was calculated as the product of diet intake (average week prior to blood sampling) and the Lys content of the corresponding diet.

Data were processed using SAS^®^ Studio (SAS Institute Inc), Metaboanalyst 5.0 software (https://www.metaboanalyst.ca/) or with STATA 14 (StataCorp, College Station, TX, USA). Graphs were drawn with GraphPad Prism 7 (version 6 for Windows, La Jolla, USA).

## Results

The plasma concentrations of the free amino acids and their metabolites at the individual timepoints and the collapsed three timepoints as well as the correlation coefficients are summarized in the Tables S2–S5 in the Supplement to this work (Supplement, Part A). At baseline, the plasma concentrations of the analytes in the rats (*n* = 95) were 376 [338–414] µM for Lys, 133 [110–152] µM for Arg, 0.62 [0.53–0.79] µM for hArg, and 3.28 [2.74–3.91] µM for GAA. Thus, the highest plasma concentration was obtained for Lys, and the lowest concentration for hArg. The molar ratios in plasma were 2.8 [2.5–3.3] for Lys/Arg, 590 [519–658] for Lys/hArg, 115 [96–139] for Lys/GAA, and 5.23 [3.93–6.64] for GAA/hArg.

We found multiple correlations between the plasma concentrations of the amino acids and some of their metabolites at baseline. Table 1 summarizes the results for Lys, hArg and GAA (see also Table S2). The highest correlation was found between hArg and Lys: *r* = 0.723, *P* < 0.0001. Neither Lys nor Arg correlated significantly with GAA. hArg correlated weakly with Arg: *r* = 0.384, *P* = 0.0001. Lys correlated also weakly with Arg: *r* = 0.387, *P* = 0.0001. The two isomeric 5-hydroxy-Lys metabolites, [5-OH-Lys(D), 5-OH-Lys(L)] correlated weakly with Lys. Monomethyllysine (MML), carboxymethyllysine (CML) and furosine are Lys metabolites formed by PTM of Lys residues in proteins. Free plasma Lys correlated weakly with MML (*r* = 0.300, *P* = 0.0031), yet not with CML or furosine. GAA correlated weakly with its precursor Gly (*r* = 0.221, *P* = 0.0314).

**Table 1:** Spearman correlations between the concentrations of free Lys, hArg, GAA and other free amino acids in the plasma of the study rats (*n* = 95) at baseline (T0)

Amino acid	Lys		hArg		GAA	
Baseline (T0)	*r*	*P* value	*r*	*P* value	*r*	*P* value
Ala	0.444	0.0000	0.197	0.0556	0.244	0.0171
Thr	0.257	0.0118	0.036	0.7275	0.324	0.0014
Gly	0.227	0.0267	0.109	0.2919	0.221	0.0314
Val	0.301	0.0031	0.088	0.3963	0.090	0.3834
Ser	0.406	< 0.0001	0.176	0.0880	0.328	0.0012
Sarcosine	0.364	0.0003	0.275	0.0070	0.031	0.7664
Leu + Ile	0.257	0.0121	0.094	0.3632	0.041	0.6915
GAA	0.172	0.0958	0.085	0.4102		
Asp + Asn	0.403	0.0001	0.225	0.0281	0.316	0.0018
OH-Pro	0.201	0.0503	0.106	0.3063	0.389	0.0001
Pro	0.446	< 0.0001	0.212	0.0394	0.272	0.0077
Met	0.404	< 0.0001	0.235	0.0217	0.382	0.0001
Glu + Gln	0.457	< 0.0001	0.255	0.0127	0.402	0.0001
5-OH-Lys (D)	0.240	0.0191	0.146	0.1593	0.143	0.1683
5-OH-Lys (L)	0.247	0.0158	0.192	0.0622	0.157	0.1284
Orn + Cit	0.276	0.0068	0.023	0.8213	0.285	0.0052
Phe	0.277	0.0066	0.132	0.2023	0.108	0.2995
Tyr	0.357	0.0004	0.154	0.1352	0.245	0.0166
**Lys**			**0.723**	** < 0.0001**	0.172	0.0958
**Arg**	**0.387**	**0.0001**	**0.384**	**0.0001**	**0.181**	**0.0789**
MML	0.300	0.0031	0.109	0.2919	0.113	0.2775
CMC	0.152	0.1415	0.211	0.0405	0.027	0.7974
**hArg**	**0.723**	** < 0.0001**			0.085	0.4102
Trp	0.183	0.0758	0.122	0.2396	0.171	0.0979
CML	0.001	0.9897	− 0.061	0.5593	− 0.106	0.3085
ADMA	0.367	0.0003	0.184	0.0744	0.078	0.4497
MMA	0.188	0.0682	0.077	0.4597	0.181	0.0799
Furosine	0.158	0.1259	0.034	0.7472	0.167	0.1060

Bold values indicate statistical significance

*GAA* guanidinoacetate, *MML* monomethyllysine, *CMC* carboxymethylcysteine, *CML* carboxymethyllysine, *ADMA* asymmetric dimethylarginine, *MMA* monomethylarginine

After 2 months, i.e., at T2, the plasma concentrations were 339 [283–392] µM for Lys and 0.85 [0.66–1.25] µM for hArg (each *n* = 81) (Table S3). After 4 months, i.e., at the third timepoint (T4), the plasma concentrations were 339 [297–396] µM for Lys and 0.96 [0.76–1.24] µM for hArg (each *n* = 77) (Table S4). One-way ANOVA revealed statistical significance for Lys (*P* = 0.0032; adjusted *P* = 0.0031 between T0 and T2) and for hArg (*P* < 0.0001; adjusted *P* < 0.0001 each for T0 and T2, and T0 and T4; adjusted *P* = 0.7665 for T2 and T4).

The plasma Lys concentration decreased on average by 12% (T0 vs. T2 and T4). The plasma hArg concentration increased on average by 17% (T0 vs. T2) and by 35% (T0 vs. T4). The plasma concentration of GAA increased from 3.28 [2.74–3.91] µM at T0 (*n* = 91) to 4.6 [3.7–5.9] µM at T2 (*n* = 81), and remained at this level at T4 (4.36 [3.48–5.62] µM; *n* = 77). The increases from T0 to T2 and from T0 to T4 were statistically significant (one-way ANOVA < 0.0001; adjusted *P* values < 0.0001, < 0.0001 and *P* = 0.2861, respectively). There was a trend to lower GAA/hArg molar ratios: 5.38 ± 1.9, 5.43 ± 2.01, and 4.78 ± 1.87 (*P* = 0.0628, one-way ANOVA), respectively.

**Fig. 2:**
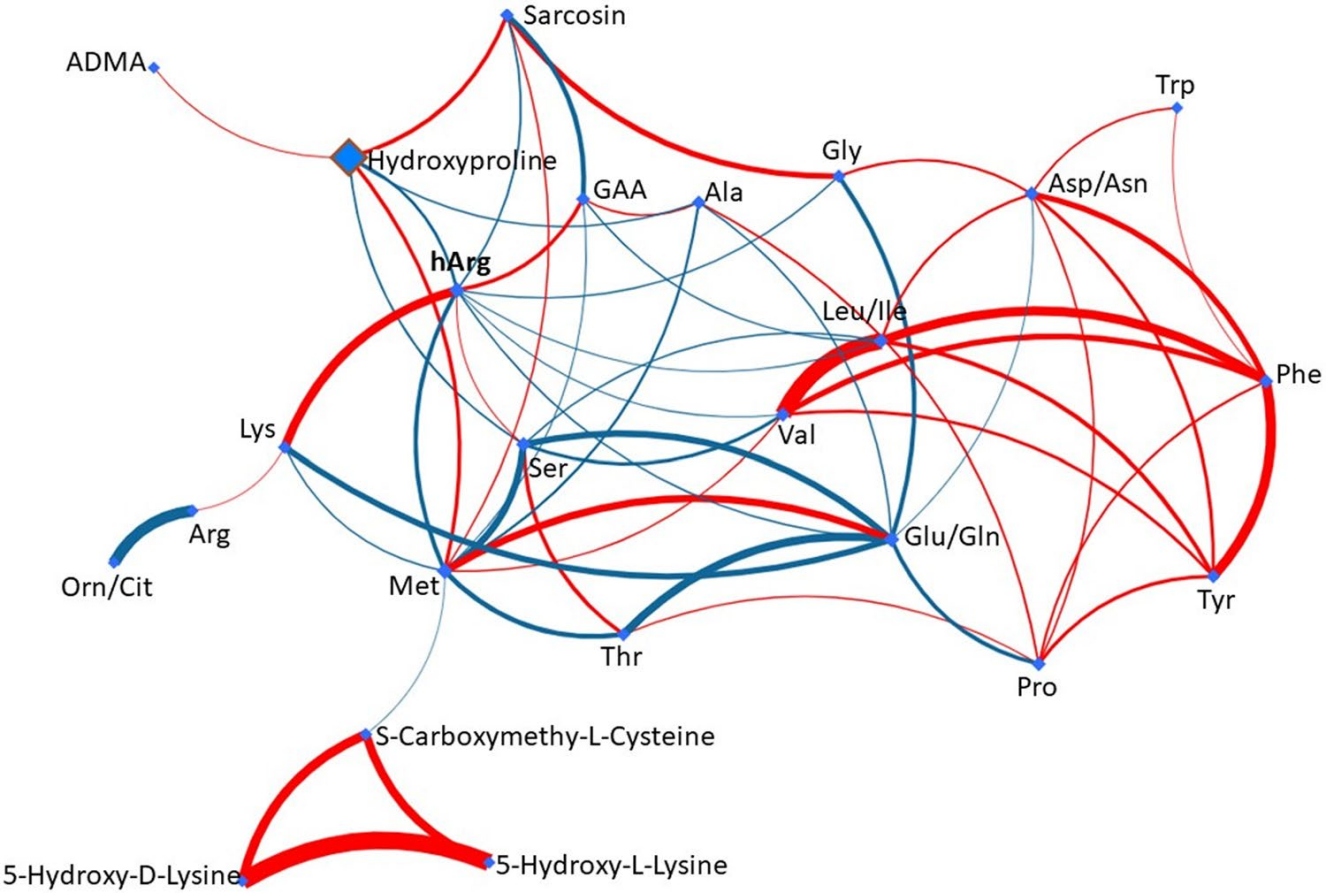
Partial correlation network between all targeted metabolites across all timepoints. The thicker the curve the stronger the correlation between two nodes. Red color means positive associations and blue color means negative associations

When considering the collapsed data from all timepoints, the plasma concentrations of Lys and GAA were significantly correlated with hArg (Table S5, Figure S1). The partial correlation network between all targeted metabolites across all timepoints is shown in Fig. 2. This figure illustrates the close correlation between Lys and hArg.

Using PLS regression, we found that four factors were required to explain most of the variation in both the predictors and the responses (Q^2^ = 57.74%, R^2^ = 71.45%). Most of the variables had VIP scores below 0.8 and small coefficients (Fig. S2). It was particularly the case for Val, Leu/Ile, Asp/Asn, Pro, Met, Glu/Gln, 5-OH-Lys(D) (Fig. S3). In contrast, Orn/Cit, Phe, MMK, Trp, ADMA, MMR and furosine were poor contributors in fitting the PLS model for predicting hArg. Finally, we found in this context that GAA and Lys were the most important analytes (Figs. S2, S3).

**Fig. 3:**
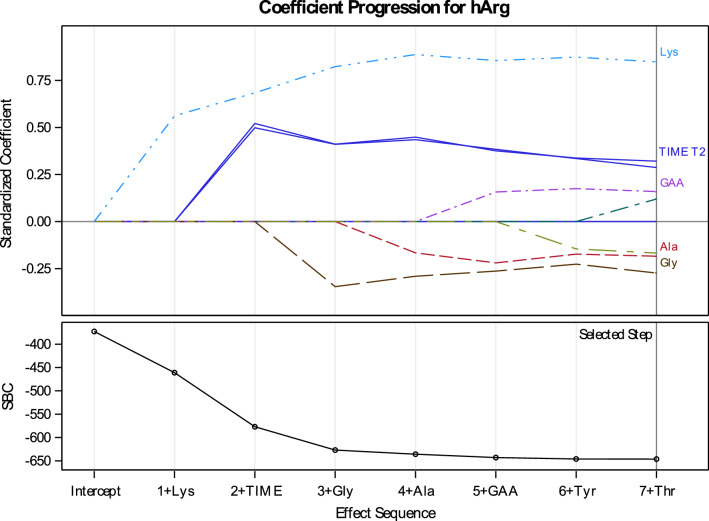
Changes in standardized estimates of coefficients of each predictor entered in the stepwise models (upper panel) and improvement in the SBC criteria (lower panel) when entering further metabolites

Using a selection algorithm, we identified a parsimonious ANOVA model for explaining hArg. Figure 3 shows the relative importance of the metabolites selected at each step in the model effects, as evaluated with their standardized coefficients and the stage when these metabolites were introduced in the stepwise model. For each model succinctly explored during model selection, Lys was found to have a large positive effect, whereas the other contributing factors, i.e., time, GAA, Ala and Gly had moderate effects on hArg.

**Fig. 4:**
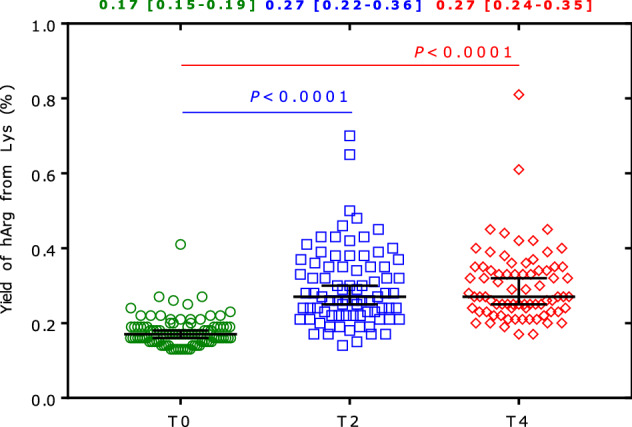
Calculated yield values for hArg from Lys in the rats of the study at baseline (T0; *n* = 95 rats), after 2 months (T2; *n* = 81 rats) and after 4 months (T4; *n* = 77 rats). One-way ANOVA and Tukey's multiple comparisons test were performed. There was no difference between T2 and T4 (*P* = 0.98). Date on the top are the median [interquartile range] values of the yield (%)

Based on the plasma concentration changes, the median yield of hArg from Lys was determined to be 0.17% at T0 and each 0.27% at T2 and T4 (Fig. 4).

In additional PLS regression analyses, the addition of diet parameters (Lys intake, protein source milk/plant and sugar/saturated fatty acids (SFA) content) as model effects did not alter the strength of the relationship between hArg, GAA and Lys (Supplement, Part B). In the selection algorithm used to identify a parsimonious ANOVA model that explain hArg with selected metabolites, we added time, Lys intake, protein source milk/plant and sugar/SFA content as class variables. The relative importance of the selected metabolites in the model was evaluated with standardized coefficients. The explanatory weight of Lys on Arg in the model was clearly higher than the weight of the different diet effects. GAA, Gly and Ala levels are still selected by the algorithm as relevant to explain variations in hArg levels. We also ran additional univariate analyses in a linear mixed model with fixed effects previously selected by the algorithm or for adjustment purposes (time, protein source, sugar/SFA level, Lys, Gly, Ala, GAA, Orn/Cit contents and Lys intake) and random (subjects). Dietary factors including Lys intake were not significant, whereas the association between hArg and Lys remained strong, significant and independent (Table 2).

**Table 2:** Mean concentrations and coefficients of variation of free amino acids and their metabolites in the study rats from the plasma concentrations measured at baseline, 2 months, 4 months

Amino acid	Mean concentration (µM)	Coefficient of variation (%)
Ala	286	1.4
Thr	365	3.7
Gly	329	7.4
Val	211	**11.5**
Ser	293	7.2
Sarcosine	1.24	**23.6**
Leu + Ile	221	**10.6**
GAA	4.08	**17.2**
Asp + Asn	81.9	**10.8**
OH-Pro	13.1	**24.5**
Pro	127	3.9
Met	82.2	9.2
Glu + Gln	953	8.3
5-OH-Lys(D)	0.3	3.3
5-OH-Lys(L)	0.93	3.3
Orn + Cit	74.6	4.8
Phe	80.2	6.9
Tyr	62.4	8.1
Lys	351	6.1
Arg	122	7.7
MML	0.64	**19.5**
CMC	1.9	6.8
**hArg**	**0.81**	**21.4**
Trp	76.6	9.4
CML	0.11	5.1
ADMA	0.44	**12.4**
MMA	0.12	8.3
Furosine	0.04	6.4

Values above 10.0 are indicated in bold

*GAA* guanidinoacetate, *MML* monomethyllysine, *CMC* carboxymethylcysteine, *CML* carboxymethyllysine, *ADMA* asymmetric dimethylarginine, *MMA* monomethylarginine

## Discussion

The plasma concentrations of many amino acids of the rats of the present study were found to correlate each other. The correlation between the plasma concentrations of Lys and hArg was the highest and greater than the correlation between Lys and Arg. Further analysis, using partial correlation network, PLS regression and stepwise regression, clearly demonstrated that hArg and Lys are closely and specifically associated over the whole set. In particular, in the whole set of variables only Lys and GAA (and to a lesser extent a few other amino acids, such as Gly) can best predict changes in plasma hArg concentrations. These observations indicate that hArg and Lys are biochemically closely related, most likely via the AGAT-catalyzed transamidination of Arg by Lys to produce hArg and Orn (Fig. 1, reaction 1). AGAT also catalyzes the formation of GAA from the transamidination of Arg by Gly (Fig. 1, reaction 2). The correlation between GAA with Gly supports this mechanism.

The results of the present study in old rats are supported by previous observations in young male rats showing increases in plasma hArg concentrations fed with Lys (Shimomura et al. 2014). The results of the present study are also supported by our previous findings in healthy young humans showing increases of the plasma Lys concentration after supplementation with hArg (Bollenbach et al. 2019). These studies indicate that hArg and Lys are mutual metabolites. Differences in correlations between the substrates of AGAT, i.e., Lys and Arg, and the products, i.e., hArg and GAA, found in the present study are likely to originate from differences in the further metabolism of hArg and GAA: hArg is a minor metabolite of AGAT, whereas GAA is a major metabolite of AGAT. In vitro, the AGAT activity with respect to GAA was found to be about 3 times higher than with respect to hArg (Watanabe et al. 1988, 1994). We assume that, both in humans and in rats, GAA is more rapidly and abundantly metabolized to creatine and further to creatinine than is hArg metabolized by decarboxylases, arginases and alanine:glyoxylate aminotransferase 2, all delivering minor metabolites (Fig. 1).

The profound increase of the plasma hArg concentration after 2 months (by 12%) and after 4 months (by 35%), as well as of GAA (by 29%) may suggest that hArg and GAA homeostasis changed during the study in slight favor of hArg. hArg and GAA are not proteinogenic amino acids. It is unlikely that proteins in the rat have contributed to hArg and GAA. Yet, our study does not allow concluding whether the changes seen in the plasma concentrations of hArg and GAA are due to the age of the rats and their non-proteinic diet, or due to elevated AGAT activity, attenuated metabolism of hArg and GAA, or impaired glomerular filtration of hArg leading to its accumulation in the blood. The mean plasma concentration of creatinine amounted to 97 µM at T0, 64 µM at T2, and 47 µM at T4. These are remarkable decreases may be due to lowered creatine synthesis from GAA. Plasma creatinine is well known to be strongly associated inversely with age including in male Wistar rats (Sanderson et al. 1997) and positively with lean mass. This may explain part of those findings. Additional factors such as cellular uptake and efflux of Arg and its derivatives including hArg and Lys, which are facilitated by specific transport proteins (Banjarnahor et al. 2020), are likely to have contributed to some of the observations of our study.

In summary, the correlations observed between the plasma concentrations of free hArg and free Lys in the rats were the highest among the free amino acids and their PTM metabolites. Endogenous hArg and Lys are mutual substrates and are primarily formed from Arg, and possibly from other guanidine compounds, via the catalytic activity of AGAT, an enzyme with a broad spectrum of substrates (Watanabe et al. 1988, 1994). Dietary Lys and hArg prevent arterial calcification in rats and mice (Shimomura et al. 2014; Rodionov et al. 2019). Present knowledge arises the question whether Lys rather than hArg supplementation may be more favorable to improve cardiovascular dysfunction resulting from impaired hArg synthesis.

## Supplementary Information

Below is the link to the electronic supplementary material.

Supplementary file1 (DOCX 2093 KB)

## Abbreviations

AGAT: Arginine:glycine amidinotransferase
AGXT2: Alanine:glyoxylate aminotransferase 2
Cit: Citrulline
CML: Carboxymethyllysine
DSPC: De-biased sparse partial correlation
GAA: Guanidinoacetate
GAMT: Guanidinoacetate *N*-methyltransferase
GC–MS: Gas chromatography–mass spectrometry
hArg: L-Homoarginine
IQR: Interquartile range
LSD: Least significant differences
MML: Monomethyllysine
NO: Nitric oxide
Orn: Ornithine
ODC: Ornithine decarboxylase
PLS: Partial least squares
PTM: Post-translational modification(s)
SAM: *S*-Adenosylmethionine
