# Supplementary material for: Lysine and homoarginine are closely interrelated metabolites in the rat
Source: Amino Acids. 2022 Mar 30;54(6):967–76. doi: 10.1007/s00726-022-03158-0 (PMC9213308; doi:10.1007/s00726-022-03158-0)
Supplement: Supplementary file 1 — Supplementary file1 (DOCX 2093 KB) [file 726_2022_3158_MOESM1_ESM.docx]

**Supplement to**

**Lysine and homoarginine are closely interrelated metabolites in the rat**

Svetlana Baskal,^1,a^ Laurianne Dimina,^2,a^ Stefanos A. Tsikas,^3^ Laurent Mosoni,^4^ Didier Remond,^4^ François Mariotti,^2,b^ Dimitrios Tsikas^1,b^

^1^ Institute of Toxicology, Core Unit Proteomics, Hannover Medical School, 30623, Hannover, Germany

^2^ Université Paris-Saclay, AgroParisTech, INRAE, UMR PNCA, 75005, Paris, France

^3^ Dean of Studies Office, Academic Controlling, Hannover Medical School, Hannover, 30623, Germany

^4^ Université Clermont Auvergne, INRAE, UMR1019, Unité Nutrition Humaine, Clermont-Ferrand, France

^a^ These authors contributed equally to the work and are both first authors

^b^ These authors contributed equally to the work and are both senior authors

**Part A: Lys, hArg, Arg and other metabolites in the study - non-consideration of diet**

Table S1: Targeted metabolites analyzed by GC-MS and corresponding chemical identification. NA, not applicable.

| **Targeted metabolite** | | **HMDB ID** | **PubChem ID** | **KEGG ID** |
| --- | --- | --- | --- | --- |
| 1 | L-Alanine | HMDB0000161 | 5950 | C00041 |
| 2 | L-Threonine | HMDB0000167 | 6288 | C00188 |
| 3 | Glycine | HMDB0000123 | 750 | C00037 |
| 4 | L-Valine | HMDB0000883 | 6287 | C00183 |
| 5 | Serine | HMDB0062263 | 5951 | C00716 |
| 6 | Sarcosine | HMDB0000271 | 1088 | C00213 |
| 7 | Leucine/Isoleucine | NA | NA | NA |
| 8 | Guanidinoacetic acid (GAA) | HMDB0000128 | 763 | C00581 |
| 9 | Aspartic Acid/Asparagine | NA | NA | NA |
| 10 | 4-Hydroxy-proline (4-OH-Pro) | HMDB0000725 | 5810 | C01157 |
| 11 | L-Proline | HMDB0000162 | 145742 | C00148 |
| 12 | L-Methionine | HMDB0000696 | 6137 | C00073 |
| 13 | Glutamic acid/Glutamine | NA | NA | NA |
| 14 | 5-Hydroxy-D-lysine (5_OH_K_D) | NA | NA | NA |
| 15 | 5-Hydroxy-L-lysine (5_OH_K_L) | HMDB0000450 | 4433 | C16741 |
| 16 | Ornithine/Citrulline | NA | NA | NA |
| 17 | L-Phenylalanine | HMDB0000159 | 6140 | C00079 |
| 18 | L-Tyrosine | HMDB0000158 | 6057 | C00082 |
| 19 | L-Lysine | HMDB0000182 | 5962 | C00047 |
| 20 | L-Arginine | HMDB0000517 | 6322 | C00062 |
| 21 | *N*^ε^-Monomethyllysine (MML) | NA | NA | NA |
| 22 | *S*-Carboxymethyl-L-cysteine (CMC) | HMDB0029415 | 1080 |  |
| 23 | Homo-L-arginine (hArg) | HMDB0000670 | 9085 | C01924 |
| 24 | L-Tryptophan | HMDB0000929 | 6305 | C00078 |
| 25 | *N*^ε^-Carboxymethyllysine (CML) | NA | NA | NA |
| 26 | Asymmetric dimethylarginine (ADMA) | HMDB0001539 | 123831 | C03626 |
| 27 | *N*^G^-Monomethylarginine (MMA) | NA | NA | NA |
| 28 | L-Furosine | HMDB0029390 | 14497053 |  |
| 29 | Nitrate | HMDB0002878 | 943 | C00244 |
| 30 | Nitrite | HMDB0002786 | 946 | C00088 |
| 31 | Creatinine | HMDB0000562 | 588 | C00791 |
| 32 | Malondialdehyde (MDA) | HMDB0006112 | 10964 | C19440 |

Table S2. Simple statistics and Pearson correlation analysis between hArg and the targeted metabolites at T0 (rats aged 16 months, n=95 for each variable). Bold characters and numbers indicate statistical significance

| **Targeted**  **metabolite** | **Mean**  **(µM)** | **SD**  **(µM)** | **Min.**  **(µM)** | **Max.**  **(µM)** | **Correlation**  **coefficient *r*** | **Prob > \|r\|**  **under H0: Rho=0** |
| --- | --- | --- | --- | --- | --- | --- |
| Ala | 286.2 | 48.3 | 172.0 | 444.0 | 0.18 | 0.090 |
| Thr | 356.6 | 58.4 | 197.0 | 475.0 | 0.15 | 0.136 |
| Gly | 364.5 | 56.7 | 230.0 | 567.0 | 0.06 | 0.584 |
| Val | 237.8 | 27.9 | 170.0 | 327.0 | 0.08 | 0.431 |
| **Ser** | 274.4 | 37.8 | 183.0 | 439.0 | 0.22 | **0.029** |
| Sarcosine | 1.64 | 0.38 | 1.08 | 3.35 | 0.12 | 0.257 |
| Leu/Ile | 247.8 | 30.5 | 177.0 | 373.0 | 0.10 | 0.316 |
| GAA | 3.38 | 0.89 | 0.87 | 5.68 | 0.02 | 0.825 |
| **Asp/Asn** | 90.4 | 13.9 | 56.4 | 118.0 | 0.22 | **0.031** |
| OH_Pro | 17.4 | 3.44 | 10.7 | 38.2 | 0.06 | 0.570 |
| **Pro** | 132.5 | 15.3 | 90.5 | 177.0 | 0.29 | **0.004** |
| **Met** | 90.6 | 7.94 | 73.9 | 113.0 | 0.24 | **0.017** |
| **Glu/Gln** | 1044.0 | 129.7 | 766.0 | 1480.0 | 0.26 | **0.010** |
| **5_OH_K_D** | 0.30 | 0.05 | 0.19 | 0.47 | 0.26 | **0.011** |
| **5_OH_K_L** | 0.96 | 0.17 | 0.61 | 1.50 | 0.29 | **0.004** |
| Orn/Cit | 87.8 | 33.1 | 35.5 | 215.0 | 0.04 | 0.727 |
| Phe | 86.5 | 9.15 | 67.2 | 111.0 | 0.11 | 0.307 |
| Tyr | 68.2 | 8.96 | 47.4 | 98.5 | 0.09 | 0.398 |
| **Lys** | 377.2 | 49.6 | 279.0 | 484.0 | **0.68** | **<0.0001** |
| **Arg** | 128.2 | 32.2 | 22.6 | 193.0 | 0.26 | **0.012** |
| MML | 0.77 | 0.10 | 0.54 | 1.00 | 0.08 | 0.433 |
| **CMC** | 1.77 | 0.43 | 0.86 | 2.98 | 0.24 | **0.021** |
| **Trp** | 83.7 | 17.2 | 19.9 | 133.0 | 0.26 | **0.012** |
| CML | 0.12 | 0.03 | 0.05 | 0.19 | 0.04 | 0.717 |
| **ADMA** | 0.50 | 0.10 | 0.26 | 0.77 | 0.24 | **0.018** |
| **MMA** | 0.13 | 0.03 | 0.06 | 0.23 | 0.24 | **0.020** |
| Furosine | 0.04 | 0.01 | 0.02 | 0.08 | 0.01 | 0.922 |
| hArg | 0.67 | 0.22 | 0.38 | 1.97 | Not applicable | |

Table S3. Simple statistics and Pearson correlation analysis between hArg and the targeted metabolites at T2 (rats aged 20 months, n=81 for each variable). Bold characters and numbers indicate statistical significance

| **Targeted**  **metabolite** | **Mean**  **(µM)** | **SD**  **(µM)** | **Min.**  **(µM)** | **Max.**  **(µM)** | **Correlation**  **coefficient *r*** | **Prob > \|r\| under**  **H0: Rho=0** |
| --- | --- | --- | --- | --- | --- | --- |
| **Ala** | 285.6 | 69.2 | 115.0 | 429.0 | 0.23 | **0.0375** |
| **Thr** | 344.8 | 77.6 | 149.0 | 512.0 | 0.41 | **0.0002** |
| Gly | 306.6 | 71.8 | 136.0 | 432.0 | 0.11 | 0.3142 |
| **Val** | 190.6 | 39.9 | 88.1 | 273.0 | 0.27 | **0.0141** |
| **Ser** | 290.2 | 60.7 | 139.0 | 425.0 | 0.36 | **0.0008** |
| Sarcosine | 1.04 | 0.26 | 0.50 | 1.83 | 0.08 | 0.4547 |
| **Leu_Ile** | 206.4 | 43.2 | 91.5 | 292.0 | 0.23 | **0.0381** |
| **GAA** | 4.89 | 1.60 | 2.20 | 9.65 | 0.54 | **<0.0001** |
| **Asp/Asn** | 78.8 | 23.9 | 26.8 | 138.0 | 0.25 | **0.0252** |
| **OH-Pro** | 11.5 | 2.81 | 5.33 | 19.9 | 0.28 | **0.0118** |
| **Pro** | 123.1 | 25.9 | 53.6 | 174.0 | 0.31 | **0.0050** |
| **Met** | 75.6 | 12.3 | 41.2 | 99.10 | 0.34 | **0.0020** |
| **Glu/Gln** | 854.8 | 202.9 | 303.0 | 1194.0 | 0.37 | **0.0007** |
| **5_OH_K_D** | 0.29 | 0.05 | 0.19 | 0.40 | 0.25 | **0.0269** |
| **5_OH_K_L** | 0.89 | 0.17 | 0.54 | 1.25 | 0.31 | **0.0047** |
| Orn/Cit | 74.5 | 28.9 | 24.1 | 170.0 | -0.05 | 0.6512 |
| Phe | 75.8 | 16.2 | 32.3 | 115.0 | 0.20 | 0.0719 |
| **Tyr** | 59.9 | 13.9 | 25.6 | 84.3 | 0.22 | **0.0435** |
| **Lys** | 340.5 | 93.0 | 128.0 | 611.0 | 0.70 | **<0.0001** |
| **Arg** | 113.4 | 38.1 | 32.6 | 200.0 | 0.50 | **<0.0001** |
| **MML** | 0.55 | 0.14 | 0.21 | 0.88 | 0.40 | **0.0002** |
| CMC | 2.01 | 0.43 | 1.22 | 3.24 | 0.16 | 0.1565 |
| **Trp** | 72.9 | 18.1 | 32.3 | 111.0 | 0.41 | **0.0002** |
| CML | 0.12 | 0.07 | 0.04 | 0.63 | -0.09 | 0.4055 |
| **ADMA** | 0.40 | 0.11 | 0.16 | 0.68 | 0.35 | **0.0015** |
| **MMA** | 0.11 | 0.04 | 0.04 | 0.24 | 0.26 | **0.0200** |
| Furosine | 0.04 | 0.01 | 0.02 | 0.08 | 0.04 | 0.7428 |
| hArg | 1.03 | 0.56 | 0.41 | 3.63 | Not applicable | |

Table S4. Simple statistics and Pearson correlation analysis between hArg and the targeted metabolites at T4 (rats aged 22 months, n=77 for each variable). Bold characters and numbers indicate statistical significance

| **Targeted**  **metabolite** | **Mean**  **(µM)** | **SD**  **(µM)** | **Min.**  **(µM)** | **Max.**  **(µM)** | **Correlation**  **coefficient *r*** | **Prob > \|r\| under**  **H0: Rho=0** |
| --- | --- | --- | --- | --- | --- | --- |
| Ala | 283.3 | 43.6 | 194.0 | 436.0 | 0.04 | 0.7230 |
| **Thr** | 386.3 | 77.9 | 202.0 | 566.0 | 0.25 | **0.0310** |
| Gly | 317.2 | 52.3 | 234.0 | 455.0 | -0.19 | 0.0910 |
| **Val** | 194.2 | 23.7 | 130.0 | 255.0 | 0.26 | **0.0226** |
| Ser | 309.3 | 56.9 | 214.0 | 466.0 | 0.00 | 0.9753 |
| Sarcosine | 1.13 | 0.22 | 0.61 | 1.70 | 0.13 | 0.2505 |
| Leu/Ile | 205.9 | 24.2 | 130.0 | 272.0 | 0.18 | 0.1133 |
| **GAA** | 4.57 | 1.47 | 1.26 | 8.13 | 0.27 | **0.0192** |
| Asp/Asn | 75.9 | 13.6 | 46.7 | 104.0 | 0.04 | 0.7127 |
| OH-Pro | 11.5 | 2.94 | 6.04 | 23.2 | 0.00 | 0.9787 |
| Pro | 122.3 | 17.5 | 85.9 | 164.0 | 0.13 | 0.2423 |
| Met | 77.6 | 6.76 | 57.7 | 91.2 | 0.21 | 0.0719 |
| Glu/Gln | 900.7 | 122.1 | 578.0 | 1128.0 | 0.20 | 0.0762 |
| 5_OH_K_D | 0.30 | 0.05 | 0.19 | 0.45 | 0.15 | 0.1940 |
| 5_OH_K_L | 0.96 | 0.23 | 0.52 | 2.22 | 0.09 | 0.4596 |
| Orn/Cit | 77.0 | 22.4 | 33.5 | 147.0 | -0.11 | 0.3313 |
| Phe | 76.2 | 8.45 | 60.7 | 96.7 | -0.01 | 0.9130 |
| Tyr | 58.1 | 8.13 | 42.0 | 81.1 | -0.12 | 0.2886 |
| **Lys** | 351.0 | 74.6 | 217.0 | 649.0 | **0.74** | **<0.0001** |
| **Arg** | 114.9 | 34.8 | 23.2 | 195.0 | 0.25 | **0.0316** |
| MML | 0.56 | 0.14 | 0.32 | 1.14 | 0.03 | 0.8198 |
| CMC | 2.00 | 0.44 | 1.21 | 3.50 | 0.06 | 0.6285 |
| Trp | 71.9 | 17.4 | 2.79 | 112.0 | 0.05 | 0.6587 |
| **CML** | 0.12 | 0.06 | 0.03 | 0.48 | -0.33 | **0.0038** |
| ADMA | 0.45 | 0.13 | 0.24 | 1.12 | 0.17 | 0.1364 |
| MMA | 0.13 | 0.04 | 0.05 | 0.23 | -0.02 | 0.8398 |
| Furosine | 0.04 | 0.02 | 0.02 | 0.22 | -0.07 | 0.5684 |
| hArg | 1.08 | 0.52 | 0.47 | 3.73 | Not applicable | |

Table S5. Simple statistics and Pearson correlation analysis between hArg and the targeted metabolites across the whole period (n=253 for each variable). Bold characters and numbers indicate statistical significance

| **Targeted**  **metabolite** | **Mean**  **(µM)** | **SD**  **(µM)** | **Min.**  **(µM)** | **Max.**  **(µM)** | **Correlation**  **coefficient *r*** | **Prob > \|r\|**  **H0: Rho=0** |
| --- | --- | --- | --- | --- | --- | --- |
| **Ala** | 285.1 | 54.4 | 115.0 | 444.0 | 0.14 | **0.0281** |
| **Thr** | 361.9 | 72.8 | 149.0 | 566.0 | 0.30 | **<0.0001** |
| **Gly** | 331.5 | 65.8 | 136.0 | 567.0 | -0.14 | **0.0227** |
| Val | 209.4 | 38.1 | 88.1 | 327.0 | -0.05 | 0.4044 |
| **Ser** | 290.1 | 53.7 | 139.0 | 466.0 | 0.27 | **<0.0001** |
| **Sarcosine** | 1.29 | 0.41 | 0.50 | 3.35 | -0.19 | **0.0024** |
| Leu/Ile | 221.8 | 39.0 | 91.5 | 373.0 | -0.05 | 0.3898 |
| **GAA** | 4.23 | 1.49 | 0.87 | 9.65 | **0.47** | **<0.0001** |
| Asp/Asn | 82.3 | 18.7 | 26.8 | 138.0 | 0.02 | 0.7262 |
| **OH-Pro** | 13.7 | 4.21 | 5.33 | 38.2 | -0.18 | **0.0033** |
| **Pro** | 126.4 | 20.4 | 53.6 | 177.0 | 0.13 | **0.0414** |
| Met | 81.8 | 11.5 | 41.2 | 113.0 | -0.02 | 0.7165 |
| Glu/Gln | 939.8 | 175.3 | 303.0 | 1480.0 | 0.06 | 0.3151 |
| **5_OH_K_D** | 0.30 | 0.05 | 0.19 | 0.47 | 0.16 | **0.0115** |
| **5_OH_K_L** | 0.94 | 0.19 | 0.52 | 2.22 | 0.15 | **0.0137** |
| **Orn/Cit** | 80.4 | 29.3 | 24.1 | 215.0 | -0.11 | 0.0695 |
| Phe | 80.0 | 12.8 | 32.3 | 115.0 | -0.05 | 0.4396 |
| Tyr | 62.5 | 11.5 | 25.6 | 98.5 | -0.07 | 0.2767 |
| **Lys** | 357.5 | 74.8 | 128.0 | 649.0 | **0.56** | **<0.0001** |
| **Arg** | 119.4 | 35.5 | 22.6 | 200.0 | 0.24 | **0.0001** |
| MML | 0.64 | 0.16 | 0.21 | 1.14 | -0.10 | 0.1120 |
| **CMC** | 1.92 | 0.45 | 0.86 | 3.50 | 0.21 | **0.0009** |
| Trp | 76.8 | 18.3 | 2.79 | 133.0 | 0.09 | 0.1466 |
| **CML** | 0.12 | 0.06 | 0.03 | 0.63 | -0.16 | **0.0095** |
| ADMA | 0.45 | 0.12 | 0.16 | 1.12 | 0.10 | 0.1006 |
| **MMA** | 0.12 | 0.04 | 0.04 | 0.24 | 0.11 | 0.0714 |
| Furosine | 0.04 | 0.02 | 0.02 | 0.22 | -0.02 | 0.7325 |
| hArg | 0.91 | 0.48 | 0.38 | 3.73 | Not applicable | |


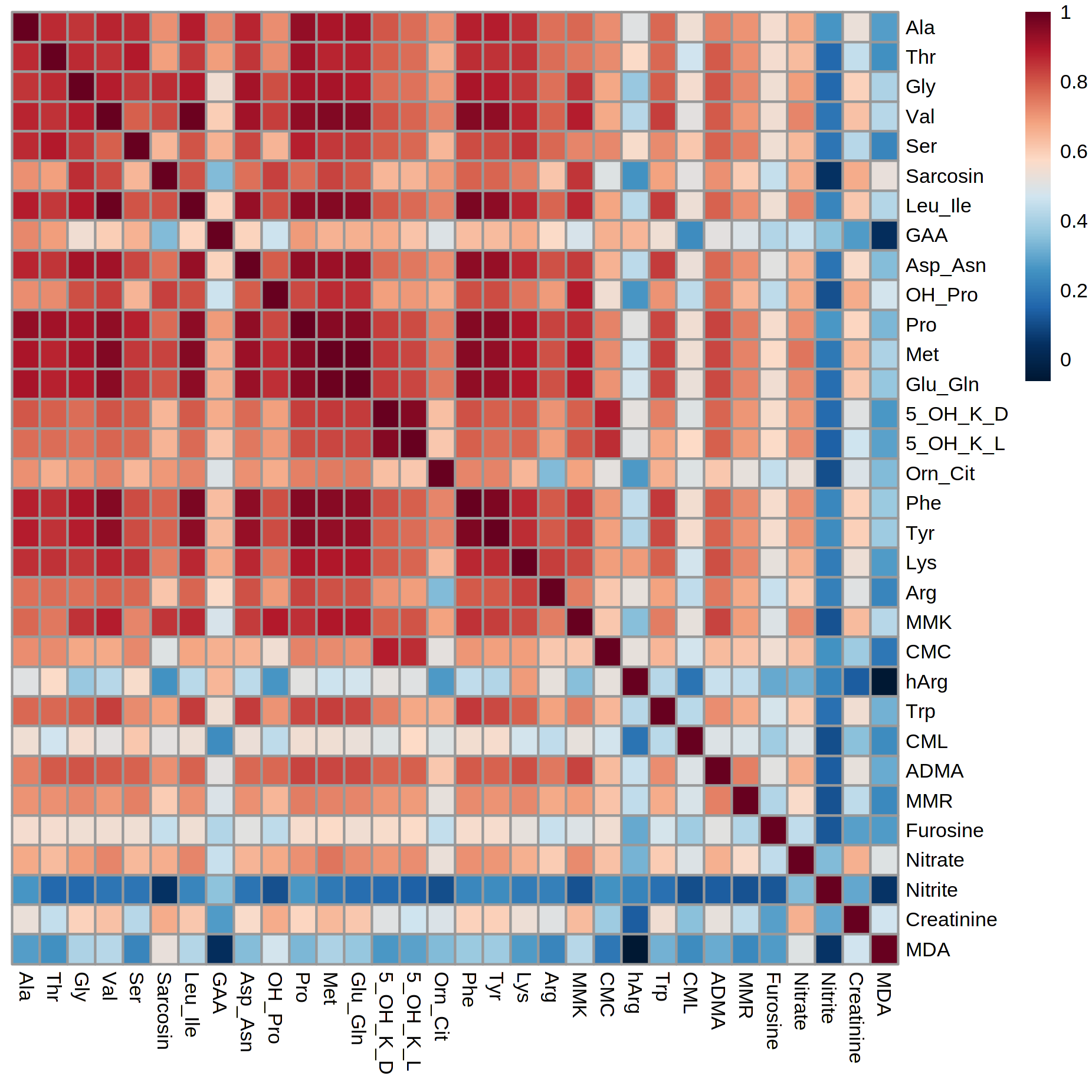
Figure S1. Overall correlation heat-map. The heat-maps reflect, in an intuitive manner, the different correlations between each of the targeted data.

Figure S2. Variable importance plot (VIP) representing the contribution of each metabolite in fitting the PLS model for both predictors and response.

Figure S3. Regression coefficients plot representing the importance each metabolite has in the prediction of just the response (i.e., hArg).

**Part B: Effects of the different diets on Lys, hArg, Arg and other metabolites**

Dietary Lys was present only “protein-bound”, but not as free lysine. The Lys content was (in mg/kg) 7200 for habituation diet, 12570 for high fat milk-based diet, 7020 for high fat plant-based diet, 10470 for standard milk-based diet and 5860 for standard plant-based diet

We considered Lys intake as a covariate in all models that have been used. Lys intake was calculated as the product of diet intake (taken as an average week prior to blood sampling) and the Lys content of the corresponding diet. We also considered the different types of diets so as to fully explore possible effect of diets.

In the PLS regression shown below, the addition of diet parameters (Lys intake, protein source milk/plant and sugar/SFA content) as model effects does not alter the strength of the relationship between hArg, GAA and Lys.


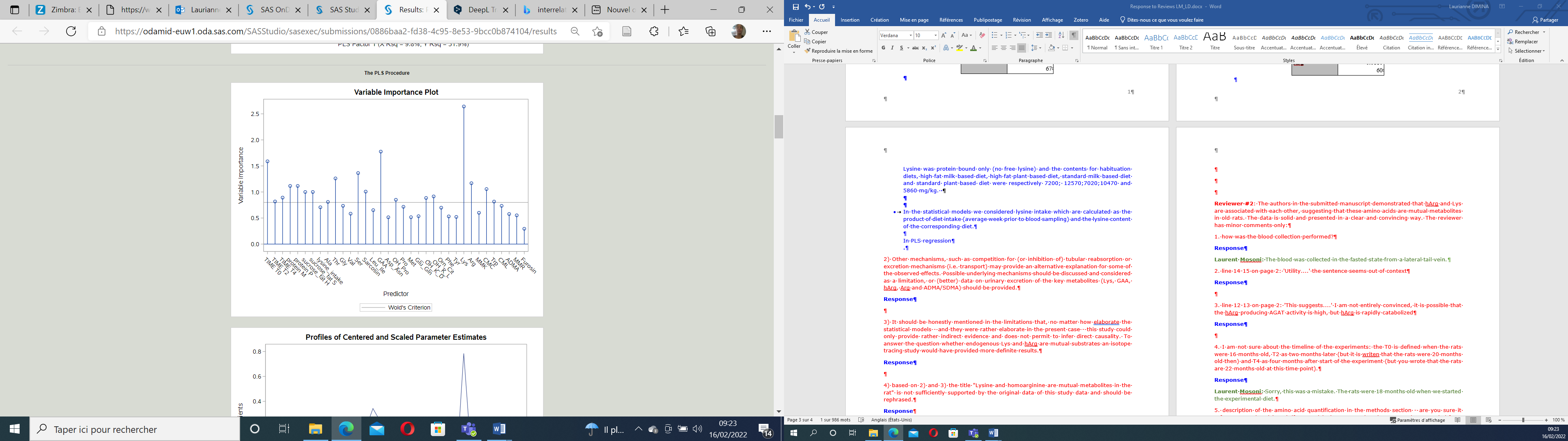


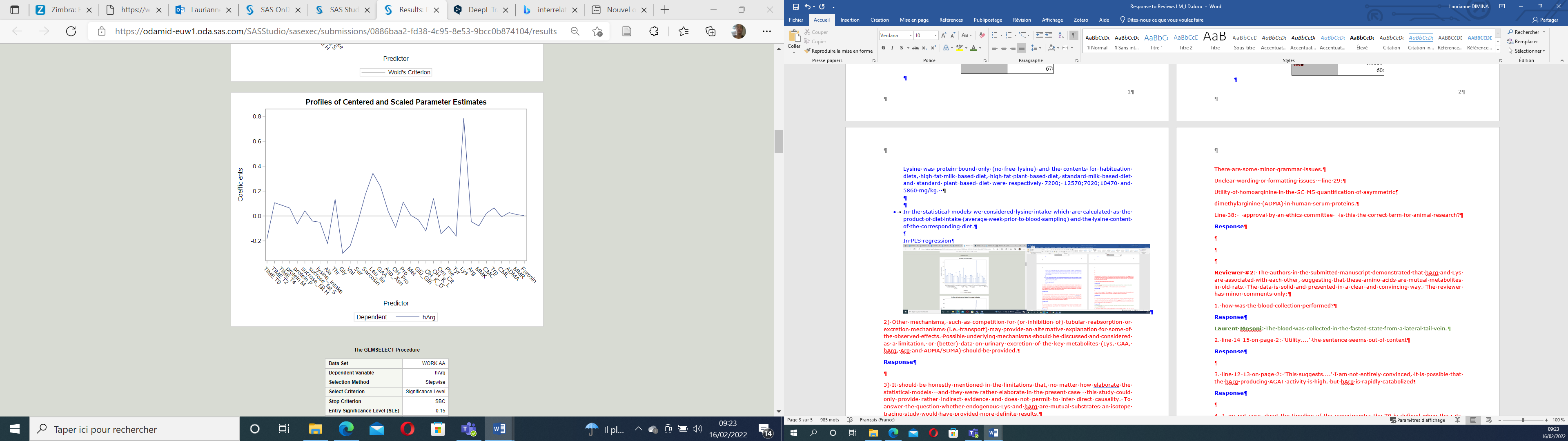


In the selection algorithm used to identify a parsimonious ANOVA model that explain hArg with selected metabolites (see below), we added time, Lys intake, protein source milk/plant and sugar/SFA content as class variables. The relative importance of the selected metabolites in the model was evaluated with the standardized coefficients. The explanatory weight of Lys on Arg in the model is clearly higher than the weight of the different diet effects. GAA, Gly and Ala levels are still selected by the algorithm as relevant to explain variations in hArg levels.


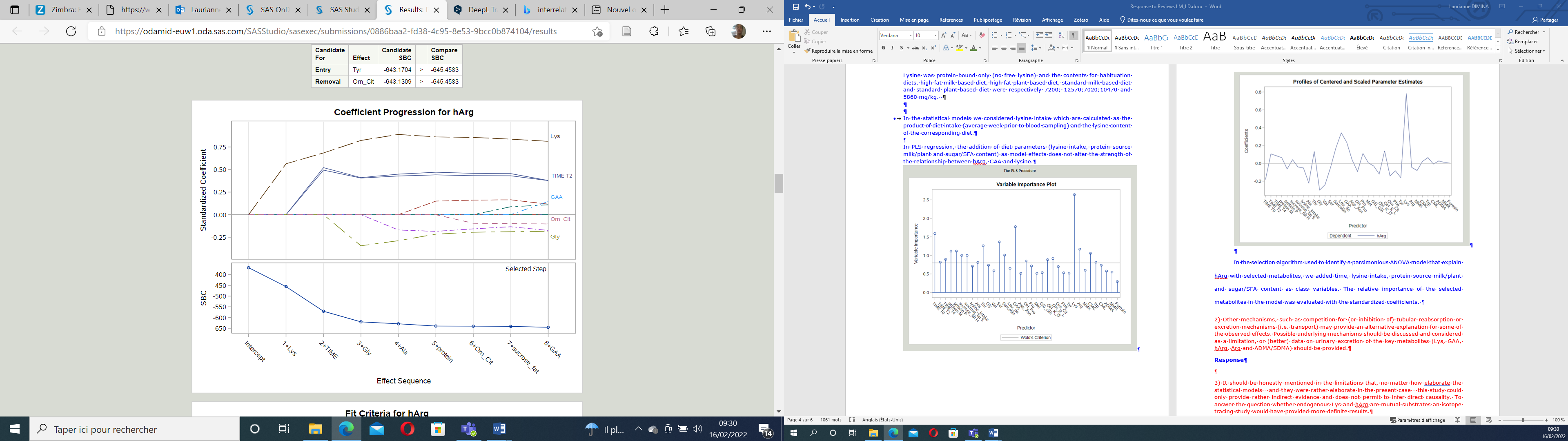


We ran additional univariate analyses in a linear mixed model with fixed effects previously selected by the algorithm or for adjustment purposes (time, protein source, sugar/SFA level, Lys, Gly, Ala, GAA, Orn/Cit contents and Lys intake) and random (subjects). Dietary factors including Lys intake were not significant, whereas the association between hArg and Lys remained strong, significant and independent as shown in the Table below.


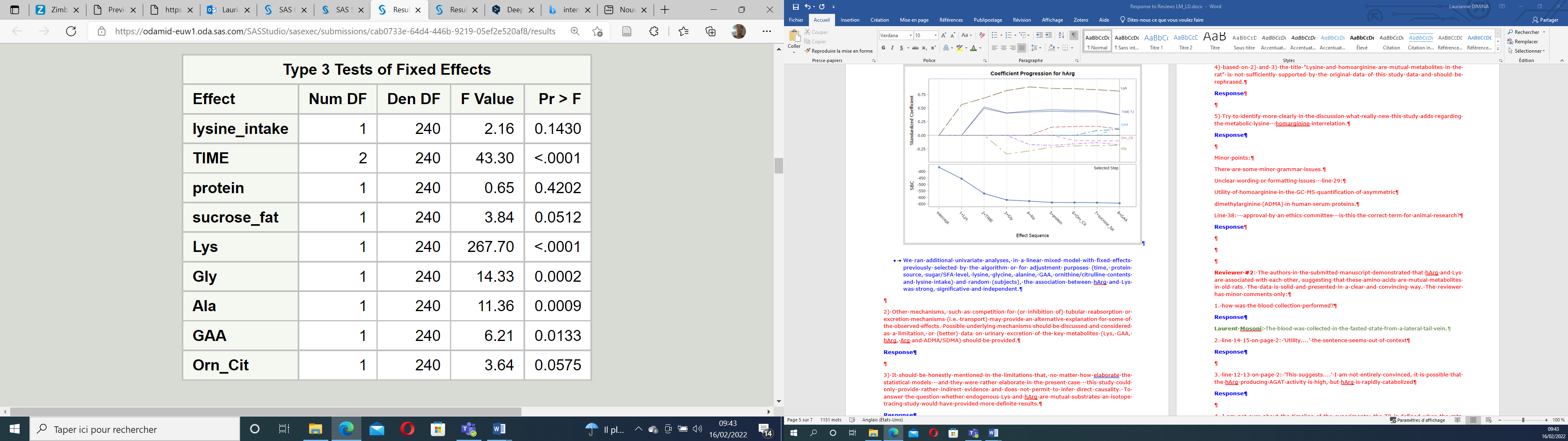


In summary, our additional analyses do not show a specific dietary modulation of the association between hArg and Lys. Accordingly, we remained focused on this relationship irrespective of other factors. In the revised manuscript, we now mention that diet parameters (such as protein, energy and Lys content) were tested in higher models but proved not significant contributors to explain variations in the relation between Lys and hArg.
